# Dr. Skipton’s Case of Twins, with Union of the Bodies of the Children

**Published:** 1842-09-03

**Authors:** 


*Case of Twins with Union of the Bodies of the Children.* By Dr. Skipton.—This was the case of a woman, about thirty years of age, and who was pregnant for the second time. On examination, after the labour had made considerable progress, one head was found protruded, and in the recess, between the chin and breast, a second was in progress of expulsion, which last was pressed so firmly on the throat of the former, as to produce great congestion, the colour of the skin changing from a reddish hue to a deep purple, and the tongue being forced beyond the lips. *Both children were expelled together;* no cry was uttered by either, nor was any movement made by them; nor could pulsation at the precordial region be detected. The mother made a good recovery. The children, both males, were united by the chest anteriorly, the union extending a few inches towards the side. The abdominal parietes of both children were deficient below the umbilicus; the viscera, having consequently no support, hung down in front. There was only one source of nourishment from the parent, and one liver, which was larger than usual. There were two hearts, *enclosed in one pericardium;* that supplying the child whose head was born first, was the smaller; the other about the normal size. Each had the usual number of hands and feet, and the organs of generation in both were perfect.—*British and Foreign Medical Review.* July, 1842. *From Dublin Med. Press,* No. 177. May 25, 1842.

